# The cortical thickness of the area PF of the left inferior parietal cortex mediates technical-reasoning skills

**DOI:** 10.1038/s41598-022-15587-8

**Published:** 2022-07-12

**Authors:** Giovanni Federico, Emanuelle Reynaud, Jordan Navarro, Mathieu Lesourd, Vivien Gaujoux, Franck Lamberton, Danièle Ibarrola, Carlo Cavaliere, Vincenzo Alfano, Marco Aiello, Marco Salvatore, Perrine Seguin, Damien Schnebelen, Maria Antonella Brandimonte, Yves Rossetti, François Osiurak

**Affiliations:** 1 IRCCS Synlab SDN, Via Emanuele Gianturco, 113, 80143 Naples, Italy; 2 Laboratory of Experimental Psychology, Suor Orsola Benincasa University, Naples, Italy; 3 Laboratoire d’Etude des Mécanismes Cognitifs (EA 3082), Université de Lyon, Lyon, France; 4 Institut Universitaire de France, Paris, France; 5 Laboratoire de recherches Intégratives en Neurosciences et Psychologie Cognitive (UR 481), Université de Bourgogne Franche-Comté, Besançon, France; 6 MSHE Ledoux, CNRS, Université de Bourgogne Franche-Comté, F-25000 Besançon, France; 7 Centre de Recherche en Neurosciences de Lyon (CRNL), Trajectoires Team (Inserm UMR_S 1028-CNRS-UMR 5292-Université de Lyon), Bron, France; 8 Mouvement et Handicap and Neuro-Immersion, Hospices Civils de Lyon et Centre de Recherche en Neurosciences de Lyon, Hôpital Henry Gabrielle, St Genis Laval, France; 9 CERMEP-Imagerie du vivant, MRI Department and CNRS UMS3453, Lyon, France; 10 Centre de Recherche en Neurosciences de Lyon (CRNL), Computation, Cognition and Neurophysiology Team (Inserm UMR_S 1028-CNRS-UMR 5292-Université de Lyon), Bron, France

**Keywords:** Cognitive neuroscience, Evolution, Psychology

## Abstract

Most recent research highlights how a specific form of causal understanding, namely technical reasoning, may support the increasing complexity of tools and techniques developed by humans over generations, i.e., the cumulative technological culture (CTC). Thus, investigating the neurocognitive foundations of technical reasoning is essential to comprehend the emergence of CTC in our lineage. Whereas functional neuroimaging evidence started to highlight the critical role of the area PF of the left inferior parietal cortex (IPC) in technical reasoning, no studies explored the links between the structural characteristics of such a brain region and technical reasoning skills. Therefore, in this study, we assessed participants’ technical-reasoning performance by using two ad-hoc psycho-technical tests; then, we extracted from participants’ 3 T T1-weighted magnetic-resonance brain images the cortical thickness (i.e., a volume-related measure which is associated with cognitive performance as reflecting the size, density, and arrangement of cells in a brain region) of all the IPC regions for both hemispheres. We found that the cortical thickness of the left area PF predicts participants’ technical-reasoning performance. Crucially, we reported no correlations between technical reasoning and the other IPC regions, possibly suggesting the specificity of the left area PF in generating technical knowledge. We discuss these findings from an evolutionary perspective, by speculating about how the evolution of parietal lobes may have supported the emergence of technical reasoning in our lineage.

## Introduction

Human technology has evolved in an unparallel way, allowing us to constantly get closer to realize our most ancestral fantasies of teleportation or telekinesis. The term *Cumulative Technological Culture* (CTC) identifies a phenomenon that describes the increase in the efficiency and complexity of tools and techniques in human populations over generations^1^. The origin of CTC constitutes a fascinating conundrum and has been considered one of the millennium’s big scientific questions^2^. CTC is prominently seen as a social phenomenon appearing from the cultural transmission of minor but incremental technological improvements over generations^3^. Accordingly, a series of social-centred cognitive mechanisms such as imitation, teaching, and theory of mind have been proposed as CTC’s foundations^4^. However, most recent research highlighted how CTC might also rely on specific, non-social, human cognitive abilities oriented toward the understanding of the physical world, namely technical reasoning^5,6^. Therefore, investigating the neurocognitive foundations of technical reasoning might be crucial to advance our understanding of how human technology has evolved^7^.

**Figure 1:**
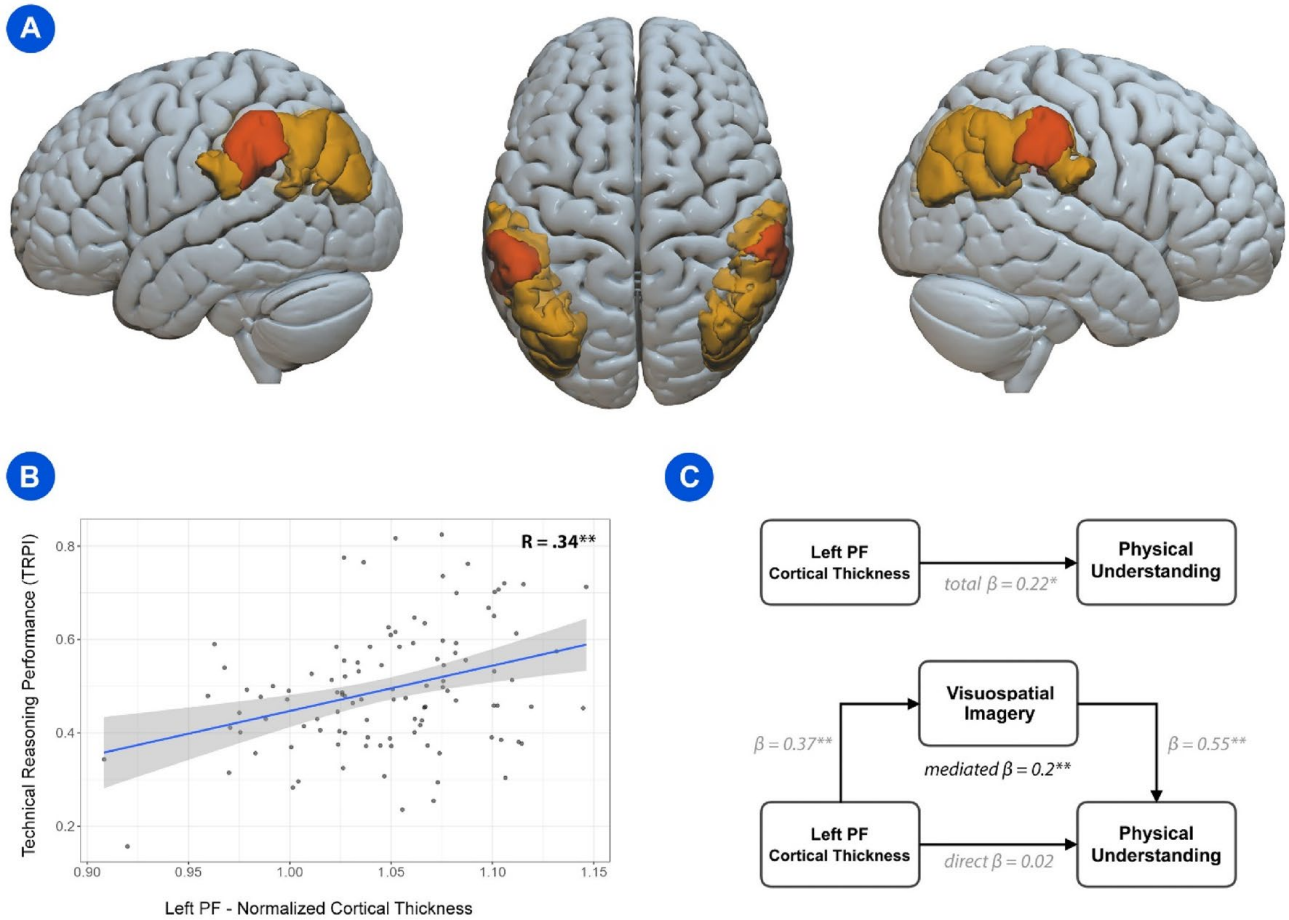
The area PF of the left inferior parietal cortex and technical-reasoning skills. (**A**) The left and right inferior parietal cortex (both highlighted in light orange), including the area PF (highlighted in orange), according to the 2016 Glasser et al.’s brain atlas^9^. The images were devised by generating a volumetric mesh with *SurfIce* (https://www.nitrc.org/projects/surfice*)*. (**B**) Pearson’s correlation between the technical reasoning performance index (TRPI) and the normalized cortical thickness of the area PF of the left IPC (R = 0.34, p < 0.001). (**C**) GLM mediation analysis, which included *Physical Understanding* as the dependent variable, *Visuospatial Imagery* as the endogenous modulator and the *Left PF Cortical Thickness* as the independent predictor (β = 0.2; p < 0.001). (**A**–**C**) *p < 0.05; **p < 0.01.

Recent functional neuroimaging evidence indicated the inferior parietal cortex (IPC; Fig. 1A) as a crucial brain locus for our aptitude to reason about the world's physical properties^8^. This brain region has been divided into ten sub-regions comprised within the supramarginal gyrus (SMG) and the angular gyrus (AG)^9^. Among these regions, most recent studies started to disentangle the specificity of the left area PF (hereafter PF), namely the largest one of the IPC, in technical-reasoning skills^8,10,11^. Neuroimaging evidence suggests how the area PF, alongside bilateral premotor cortex and superior parietal lobes, can be selectively recruited when individuals reason about physical events (i.e., physical tasks and physical interactions) compared to non-physical events (i.e., color task and social interactions)^12^. In addition, brain-lesions studies coming from the clinical neuropsychological domain, underline how familiar and novel tool use/making may be dramatically impaired after damage to the area PF, hence highlighting its key role in the ability to technically reason about mechanical tool-use actions^13–18^. Whereas converging evidence on the role of the area PF in technical reasoning begins to accumulate in the literature, to the best of our knowledge, no studies directly investigated the relations between its structural characteristics and individuals’ technical-reasoning performance. Nonetheless, studying the associations between brain volume measures and cognitive functions has been a long-standing research interest in both clinical and neurotypical populations^19–21^. Indeed, the cortex may be seen as a narrowly folded sheet of neurons that ranges in thickness between 1.5 and 4.5 mm^22^. Thus, in structural brain-imaging research, a common volume-related measure used is the cortical thickness as it reflects the size, density, and arrangement of cells and might be associated with cognitive performance^23–25^. For example, differences in cortical thickness of distinct brain areas have been correlated with intelligence in humans^23^ and even in chimpanzees^26^, as well as with total cognition^27^, musicianship and absolute pitch ^28^, executive functions^29^, memory^30^, attention^31^, language abilities^32^, and relational reasoning (i.e., the ability to consider relationships between multiple mental representations)^33^.

In the present study, we explored the structural correlates of technical reasoning, hypothesizing that cortical thickness of the area PF may be critically associated with such skills. To test this hypothesis, we assessed participants' technical-reasoning skills using two psycho-technical subtests extracted from the NV7 battery^34^. These subtests aimed at evaluating the different aspects of technical reasoning, namely physical world’s understanding and visuospatial imagery. We repeatedly found that the scores obtained on both these subtests are the best predictor of cumulative performance in micro-society experiments, in which participants had to either build a paper airplane or a tower with ten wires^35–37^. This link was not found, for example, with fluid intelligence (e.g., Raven’s Matrices) or creativity tests (e.g., alternative object use)^36^, thus confirming how the cognitive abilities assessed by these subtests are strongly related to technical reasoning. Therefore, we first assessed participants’ technical-reasoning performance by using the two psycho-technical tests; then, we extracted the cortical thickness of each IPC's area (i.e., PGp, PGs, PGi, PFm, PF, PFt, PFop, IP0, IP1, and IP2)^9^ for both the left and the right hemisphere from the participants' 3 T T1-weighted magnetic-resonance brain images we acquired. Finally, we analysed the relations between the cortical thicknesses of the IPC's brain regions and the psycho-technical scores, positing that the area PF’s cortical thickness were the only significant measure in predicting participants' scores.

## Results

As a first exploratory analysis, we implemented a forward stepwise regression model, including the normalized cortical thicknesses of the ten sub-regions (i.e., PGp, PGs, PGi, PFm, PF, PFt, PFop, IP0, IP1, and IP2)^9^ of both the left and right IPC as possible predictors of the NV7 scores, taken individually as outcome variables (M_Physical_Understanding_ = 9.84, SD_Physical_Understanding_ = 3.37; M_Visuospatial_Imagery_ = 22; SD_Visuospatial_Imagery_ = 5.98). At each step, we maintained only the regressors that had a p-value < 0.05, discarding the others from the model. We found that only the cortical thickness of the left area PF explained both the physical-understanding score (p < 0.05) and the visuospatial score (p < 0.001), while the cortical thickness of the right area PF explained the visuospatial score only (p < 0.05). Consequently, we calculated a series of Pearson’s correlations among the two NV7 scores and the cortical thicknesses of the left and right area PF. We found a significant, positive strong correlation between the two psycho-technical scores (r = 0.56; p < 0.001). Also, we found three significant positive correlations between the left PF cortical thickness and both scores (r_Physical_Understanding_ = 0.23, p_Physical_Understanding_ < 0.05; r_Visuospatial_Imagery_ = 0.37, p_Visuospatial_Imagery_ < 0.001), and between the right PF cortical thickness and the visuospatial-imagery score (r_Visuospatial_Imagery_ = 0.21, p_Visuospatial_Imagery_ < 0.05). Based on the strong association between the two NV7 scores (r = 0.56; p < 0.001), to summarize participants’ technical performance in a single discrete measure, we constructed a technical-reasoning performance index (TRPI) by standardizing the NV7 scores at the participant’s level (see “Methods”). We found a significant positive Pearson’s correlation between left PF cortical thickness and TRPI (r_TRPI_ = 0.34, p_TRPI_ < 0.001; Fig. 1B). We performed a linear regression to investigate the nature of the relationship, which showed a significant linear trend (R^2^ = 0.12; p < 0.001). Finally, to further investigate the relationships between the two psycho-technical scores and the left area PF, we speculated that visuospatial-imagery skills might be a kind of prototypical ability lying at the root of physical understanding, both mediated by the left PF cortical thickness. Therefore, we devised a GLM mediation analysis including *Physical Understanding* as dependent variable, *Visuospatial Imagery* as the endogenous modulator and the *Left PF Cortical Thickness* as the independent predictor. We found that the visuospatial-imagery score fully mediated the relationship between the left PF cortical thickness and the physical-understanding score (β = 0.2; p < 0.01; bootstrapped unstandardized mediated indirect effect 14.93; 95% CI 6.2–25.02; Fig. 1C, Table 1).

**Table 1:** GLM mediation analysis.

Type	Effect	Estimate	SE	95% CI		Beta	z	p
				Lower	Upper			
Indirect	Left PF CT → Visuospatial Imagery → Physical Understanding	14.93	4.77	6.21	25.02	0.20	3.13	0.002
Component	Left PF CT → Visuospatial Imagery	47.85	12.77	21.94	73.58	0.37	3.75	< .001
	Visuospatial Imagery → Physical Understanding	0.31	0.05	0.21	0.41	0.55	6.49	< .001
Direct	Left PF CT → Physical Understanding	1.46	4.95	-9.35	10.92	0.02	0.29	< .001
Total	Left PF CT → Physical Understanding	16.38	6.87	2.92	29.84	0.22	2.39	0.017

The GLM mediation model^63^ included the *Physical Understanding* score (Physical Understanding) as the dependent variable, the *Visuospatial Imagery* score (Visuospatial Imagery) as the endogenous modulator and the *Left PF Cortical Thickness* (Left PF CT) as the independent predictor. Confidence Intervals were calculated by using bootstrap procedures^64^. Unstandardized mediated effects (ME) for each of 10,000 bootstrapped samples were computed. Then, the MEs at 2.5th and 97.5th percentiles were determined. The reported betas are completely standardized effect sizes^66^.

## Discussion

The main finding provided by our study concerns the individuation of the area PF of the left IPC as a potential structural correlate of technical reasoning. Indeed, our results indicate that psycho-technical performance is associated with the cortical thickness of such a relatively newly investigated IPC’s brain region. In addition, we found that visuospatial-imagery skills fully mediated the association between physical understanding and the cortical thickness of the left area PF. This result is consistent with the idea that technical reasoning might be seen as a cognitive process emerging by adaptation from visuospatial-imagery skills. In this sense, technical reasoning might represent an evolutionary leap, a perspective that might find room in the paleontological debate about the characterizing and species-specific evolution of the *Homo Sapiens*’ parietal cortex^38,39^. In this context, the human evolution of parietal lobes may support the emergence of technical reasoning in our lineage, thus constituting an important evolutionary step through which technology may have evolved^40^. This evolutionary perspective may imply a different degree of brain lateralization between visuospatial-imagery (less lateralised) and physical-understanding (more lateralised)^41^. Such a theoretical speculation might gain partial support by our preliminary results, which suggest the potential involvement of both the areas PF in visuospatial-imagery skills and of only the left area PF in physical-understanding skills. However, future studies across species and investigations involving *reinforcement learning* as well as other structural measurements (e.g., cytoarchitecture) should test these predictions.

Surprisingly, technical reasoning entered the CTC’s debate only recently^5^. Indeed, as we introduced above, CTC has been traditionally explained as a phenomenon resulting from human social skills, allowing individuals to be unique social learners^4^. Nevertheless, micro-society studies have shown how improvements of a physical system over generations are accompanied by an increase of the understanding of the system^5,42^ and are critically predicted by learners’ technical-reasoning skills, assessed through the same psycho-technical tests we used in this study^37^. Those improvements may also occur in reverse-engineering conditions, in which participants cannot interact with each other at all (i.e., no possibility of imitation or teaching). However, the findings we report here do not exclude the critical role of social learning in CTC as an irreplaceable source of technical inspiration, nor they rule out the importance of more elaborated forms of social learning (e.g., teaching) for the transmission of technical content. Instead, they further underline the involvement of technical-reasoning skills in CTC.

Technical reasoning has been considered a prerogative skill in human tool use and tool making^43–45^. These abilities are at the root of the human proclivity for materiality and might be assumed as fundamental CTC’s cornerstones. Increasing evidence from cognitive neuroscience indicates a wide set of fronto-temporo-parietal networks as neural correlates of tool use/making^8,46^. Within these networks, IPC seems to be critically implicated in tool use/making activities^17,47^. Interestingly, patients with IPC lesions may encounter difficulties in using familiar tools (e.g., hammer), as well as in solving such technical problems as selecting, using, and making novel tools^43,48,49^. Transversally, in line with the above-discussed evolutionary perspective, neuro-archaeological research emphasized how the acquisition of tool-making abilities is correlated with the structural remodelling of the parietal regions^50^. Also, alongside middle-temporal and frontal areas, IPC is critically involved in integrating multiple information modalities (e.g., somatosensory, visual, auditory, and semantic) to generate representations usable in everyday-life technical problem-solving activities^51–56^.

The bulk of extant findings we summarized above fits with the idea that a common cognitive process, namely technical reasoning, might be involved in all the different manifestations of human materiality. However, embracing the concept of technical reasoning within the wider gnoseological horizon of cognitive science represents only the first step to expand our understanding of CTC’s foundations. The second step is detailing technical reasoning’s neural correlates. Thus, by identifying a direct structural link between a specific brain region, namely the area PF of the left inferior parietal cortex, and technical-reasoning skills, the present study adds an informative piece to the puzzle of the foundations of human technical mind. Hopefully, future theories may find in our results a hint to construct an interdisciplinary debate about technology evolution as well as on the cognitive abilities associated with it.

## Methods

### Participants

116 right-handed participants (70 females; mean age = 23.9 years, SD 3.9) volunteered for participating in the study. All participants were Lyon’s university students. Participants’ recruitment criteria were the following: (i) lack of current or past history of alcohol/drug abuse; (ii) lack of current or past history of major psychiatric illnesses; (iii) lack of history of brain injury, stroke, or any other major clinical condition; (iv) lack of current or past use of psychoactive medications. Such criteria were assessed through a clinical interview performed by an expert medical doctor. Handedness was assessed by using the *Edinburgh Handedness Inventory* (EHI)^57^. Eight participants (4 females) were excluded from the analyses due to their EHI score < 50. All participants gave written informed consent on their participation to the study.

### Psycho-technical assessment

**Figure 2:**
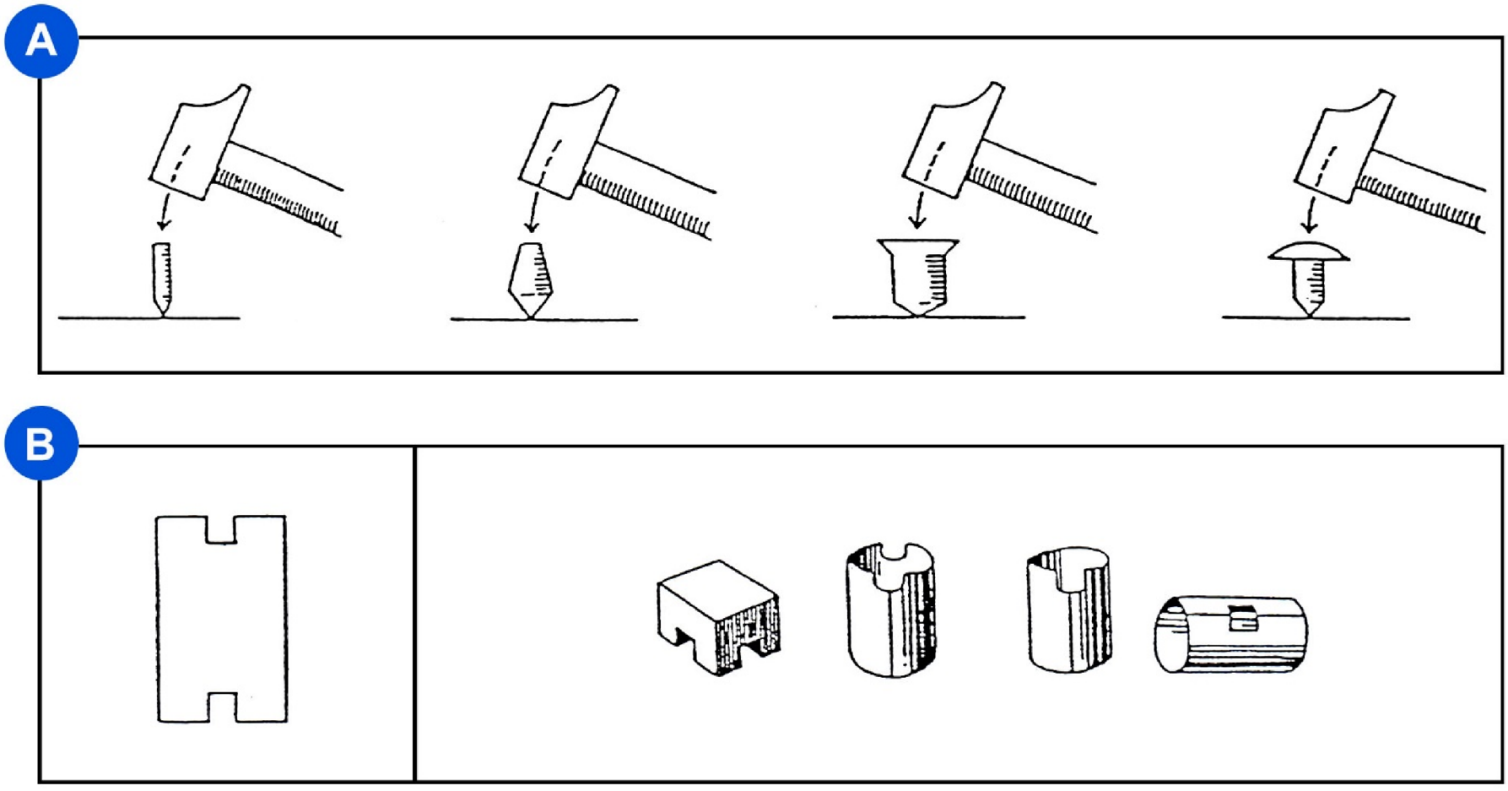
Psycho-technical assessment. (**A**) One of the 24 items we used to evaluate participants’ understanding of physical properties. In the example depicted, participants were asked to select which of the four nails were hammered more easily. (**B**) One of the 38 items we used to evaluate participants’ visuospatial-imagery skills. Participants were asked to identify which of the four 3D figures showed on the right corresponded to the 2D pattern on the left. Both the subtests were extracted from the NV7 battery^34^.

In this study, two subtests of the pencil-and-paper NV7 psycho-technical battery were used to evaluate different degrees of participants’ technical-reasoning skills^34^. The first subtest, which included 24 items, aimed at evaluating participants’ understanding of physical properties (e.g., selecting among four different nails, the easiest one to hammer; Fig. 2A). The second subtest, which comprised 38 items, aimed at measuring participants’ visuospatial-imagery skills. The subtest required participants to select among four three-dimensional geometrical shapes the one corresponding to a specific two-dimensional pattern (Fig. 2B). The NV7 battery is a multifactorial assessment battery that was designed to help psychologists in the professional orientation of young people with a low level of education (e.g., early school leaving, school failure). It consists of ten subtests (e.g., physical reasoning, visuospatial imagery, attention, arithmetic operations, arithmetic problems, verbal comprehension, orthography, deductive reasoning, inductive reasoning, and analogical reasoning). The battery’s psychometric properties were examined in a sample of 867 individuals aged between 16 and 25 (355 females) and were found to be satisfactory (e.g., reliability for each subtest: KR20 scores ranged from 0.74 to 0.97; even–odd correlations ranged from 0.57 to 0.97). The structural and content validity of the battery was confirmed by a three-factor model explaining 72.1% of variance, with a first factor reflecting non-verbal reasoning, a second verbal/cultural knowledge, and a third cognitive resources. This model was very close to the classical two-factor model of intelligence battery (i.e., non-verbal/fluid cognition versus verbal/crystallized cognition). The battery was also standardized on another sample of 300 individuals aged between 16 and 25 and with a low level of education (150 females). More information about the NV7 battery is available on the Pearson Clinical’s website (https://www.pearsonclinical.fr/nv7).

### Procedure

The study has been conducted in the Laboratory for the Study of Cognitive Mechanisms (EA 3082) at University of Lyon (Lyon, France) and in the Lyon’s Neuroimaging Department (CERMEP, Lyon France). All the experimental procedures followed the ethical standards laid down in the 1964 Declaration of Helsinki. The study received the approval from the French Ethics Committees (approval number: 2018-A00734-51 and 2019-A00646-51). Participants were randomly recruited through advertisements posted on social media websites. One week before the MRI session, participants signed the informed consent to take part in the study and were seen by a medical doctor to ascertain their eligibility for the MRI session. Then, they completed the two psycho-technical subtests of the NV7 battery in the Laboratory for the Study of Cognitive Mechanisms (EA 3082) at University of Lyon (Lyon, France). The maximum duration of each test was 5 minutes. Each score was calculated as the number of correct answers given in the 5-min time interval. One week later, participants were admitted to the Lyon’s Neuroimaging Department (CERMEP, Lyon France) for the MRI session.

### MRI scanning and brain morphometry analysis

Three-dimensional (3D) T1-weighted (T1w) sequences (TR = 3000 ms, TE = 2.93 ms, flip angle = 8°, voxel-size = 0.8 × 0.8 × 0.8 mm, matrix 280 × 320, field of view 224 × 256) were collected in DICOM format by using a 3-Tesla MR Siemens Prisma scanner with a 64-channel head coil. Anonymized DICOM imaging data of T1w structural images were converted to nifti format using the *dcm2niix* software^58^. All data were visually inspected for quality assurance prior to analyses to check for major visible artifacts and incidental brain abnormalities by an experienced neuroradiologist*.* Then, we extracted no-reference image quality metrics from structural T1w images by using MRIQC (see Supplementary Fig. SM1 in Supplementary Material 1)^59^. We chose the *Human Connectome Project Multi-Modal Parcellation* atlas (version 1.0; HCP-MMP1)^9^ to obtain an adequate number of cortical parcels (i.e., 180 areas per hemisphere), hence including the areas of the PF complex within the supramarginal gyrus, alongside the other inferior parietal lobe’s areas of the angular gyrus. Such an atlas included the following left and right inferior parietal lobe’s areas: PGp, PGs, PGi, PFm, PF, PFt, PFop, IP0, IP1, and IP2. To perform morphometric analysis of these regions, brain surfaces of each subject were reconstructed using the *recon-all* procedure (FreeSurfer 6)^60^, according to the following elaboration processes: (i) spatial inhomogeneity correction; (ii) non-linear noise-reduction; (iii) skull-stripping; (iv) subcortical segmentation; (v) intensity normalization; (vi) surface generation; (vii) topology correction; (viii) surface inflation; (ix) registration to a spherical atlas; (x) cortical thickness calculation. To estimate cortical thickness of the parcels of interest, the HCP-MMP1.0 atlas was first projected onto the *FreeSurfer* reference space (i.e., *fsaverage*) and then taken to the subject's native space with a sequence of *FreeSurfer* commands (i.e., *mri_annotation2label*, *mri_label2label*, *mri_label2annot*, *mris_label2annot*). Volume-registered analyses of the brain cortex in MNI space are widely used in the literature^61^. Thus, we followed a fully reproducible approach based on traditional volume-based analysis to generate cortical surfaces. One may argue that such an approach might be suboptimal when using the HCP-MMP1.0 parcellation^62^. However, it should be noted that any potential new sources of variance related to realignment processes would affect all the brain areas and not only the PF ones. Nevertheless, we found significant correlations concerning only the left and right area PF. The segmentation results were visually inspected prior to the volume and thickness analysis to confirm that no major errors were present. No manual edits were done. After the previous steps, anatomical information for each cortical region, including cortical thickness, has been carried out with a specific *FreeSurfer* command (i.e., *mris_anatomical_stats*). All analyses ran on a GNU/Linux workstation (i.e., GNU/Linux Centos 7).

### Statistical analyses

In this study, several hypothesis-driven statistical data analyses were implemented. To assess the normal distribution of participants’ cortical thicknesses and NV7 scores, multiple Shapiro–Wilk test were performed. We normalized participants’ cortical thicknesses by considering the whole-brain mean cortical thickness, thus considering all the HCP-MMP1 parcellations for both hemispheres, at the participant level (see Supplementary Results for non-normalized analyses). The first exploratory analysis comprised a forward stepwise regression model to identify possible predictors of the NV7 scores among the cortical thicknesses (CT) of both the left and right IPC areas (10 areas per hemisphere). By including, along with the area PF, all the other IPC areas as potential predictors in the stepwise model, we adopted a strategy aimed at highlighting that, in line with our experimental hypothesis, only the cortical thickness of the area PF, and not of the other IPC regions, can be associated with technical reasoning. Also, while we could have included random regions such as in the occipital or the prefrontal areas, that we know have no link with technical reasoning, we preferred to include regions that are very close to the PF, thus decreasing our chance to obtain a specific result for PF. Hence, we chose the worst experimental scenario with respect to our experimental hypothesis. At each step, variables were chosen based on p-values (p < 0.05). Then, a series of Pearson’s correlations were performed: (i) between the two participants’ NV7 scores; (ii) between the two NV7 scores and the predictors identified by the regression model, namely the participants’ left and right area PF of the IPC. Given the strong association between the two NV7 subtests, a post-hoc technical-reasoning performance index (TRPI) was constructed to summarize participants’ technical performance in a single discrete measure. To do that, both the technical-reasoning scores were normalized at the participant level. Therefore, for each participant, the TRPI was calculated according to the following equation:

$$TRPI= \frac{\frac{{x}_{score 1}}{{max}_{score 1}} + \frac{{x}_{score 2}}{{max}_{score 2}}}{2},$$

where ${x}_{score 1}$ and ${x}_{score 2}$ were the participants’ scores (i.e., physical-understanding and visuospatial-imagery score, respectively); ${max}_{score 1}$ and ${max}_{score 2}$ were the maximum-obtainable scores at the two NV7 subtests (i.e., 24 points for the physical-understanding skills and 38 points for the visuospatial-imagery skills). The TRPI varied between 0 and 1, where 0 were the lower technical-reasoning performers and 1 the higher ones (M_TRPI_ = 0.49; SD_TRPI_ = 0.13). An additional Pearson correlation was calculated between left PF CT and TRPI. Then, a linear regression between left PF CT and TRPI was performed to better characterize the association between these two variables (but see Supplementary Results for the cross-validation of a stepwise linear model including all the twenty right and left IPC regions). No effects of age and gender on technical reasoning skills are reported in literature. However, we checked the absence of intervening factors by using an ad-hoc GLM. We found no effects of gender and age in predicting participants’ technical-reasoning performance (see Supplementary Results). To further explore the interrelationships between the two NV7 scores and the cortical thickness of the left PF area, a GLM mediation analysis was modelled^63^. Such a mediation analyses included the first NV7 score (i.e., physical-understanding skills) as the dependent variable, the second NV7 score (i.e., visuospatial-imagery skills) as the endogenous modulator and the cortical thickness of the left PF area as the independent predictor^63^. To test the significance of the mediated effect (ME), bootstrapping procedures were used. Within the context of a GLM mediation model, bootstrap procedures^64^ provides a more sensitive test for assessing the magnitude of indirect effects than the standard Sobel test^65^. Hence, two-tailed p-values were calculated from the bootstrap confidence interval. Unstandardized mediated effects for each of 10,000 bootstrapped samples were computed, and the 95% CI was calculated by determining the MEs at 2.5th and 97.5th percentiles. Finally, to verify the soundness of our findings concerning the role of the cortical thickness of the area PF in predicting technical-reasoning performance, we implemented a whole-brain descriptive analysis (see Supplementary Results). To perform the GLM mediation analysis we used the lavaan^66^, namely an R package for structural equation modeling. All statistical analyses were performed by using R (v.4.0.2; https://www.r-project.org) and/or the Jamovi statistical software (v.2; https://www.jamovi.org). An alpha level of 0.05 was used for all the statistical analyses.

## Supplementary Information

Supplementary Information 1.

Supplementary Information 2.

## Data Availability

The data that support the findings of the present study are available at https://osf.io/thu74.
