# Supplementary material for: The cortical thickness of the area PF of the left inferior parietal cortex mediates technical-reasoning skills
Source: Sci Rep. 2022 Jul 12;12:11840. doi: 10.1038/s41598-022-15587-8 (PMC9276675; doi:10.1038/s41598-022-15587-8)
Supplement: Supplementary file 1 — Supplementary Information 1. [file 41598_2022_15587_MOESM1_ESM.pdf]

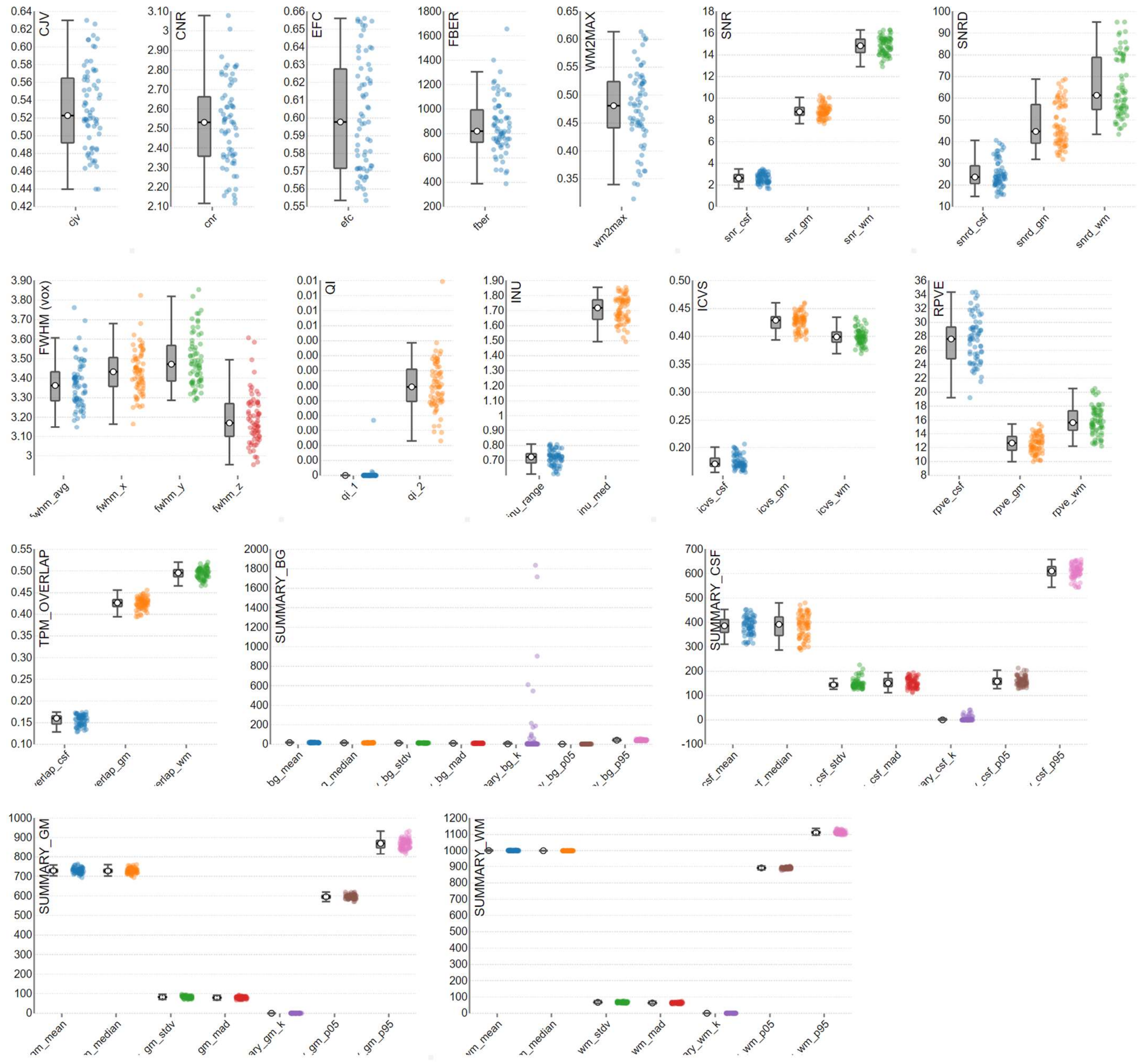

Figure SM1. MRIQC Summary

Report generated by MRIQC (version 0.16.1) related to quality metrics from the study's structural T1w images. Details and specifications concerning the metrics depicted here are available online at *https://mriqc.readthedocs.io.*
