# Supplementary material for: The cortical thickness of the area PF of the left inferior parietal cortex mediates technical-reasoning skills
Source: Sci Rep. 2022 Jul 12;12:11840. doi: 10.1038/s41598-022-15587-8 (PMC9276675; doi:10.1038/s41598-022-15587-8)
Supplement: Supplementary file 2 — Supplementary Information 2. [file 41598_2022_15587_MOESM2_ESM.pdf]

Supplementary Results

The cortical thickness of the area PF of the left inferior parietal cortex mediates technical-reasoning skills

Federico *et al.* (2022)

1. Replication of the primary statistical analyses with non-normalized data

We replicated the forward stepwise regression model presented in the article, with the non-normalized cortical thicknesses of all the ten areas of the left and right inferior parietal cortex (IPC)[1] as possible predictors of the NV7[2] scores, taken individually as outcome variables. Step by step, we maintained only the regressors that had a p-value < 0.05. We found that only the non-normalized cortical thickness of the left area PF explained both the physical-understanding ($p < 0.05$) and the visuospatial-imagery score ($p < 0.01$). We calculated a series of Pearson's correlations among the NV7 scores and the cortical thicknesses of the left area PF. We found significant positive correlations between the left PF cortical thickness and both scores ($r_{Physical\_understanding} = 0.20$, $p_{Physical\_understanding} < 0.05$; $r_{Visuospatial\_Imagery} = 0.29$, $p_{Visuospatial\_Imagery} < .01$). Finally, we found a significant positive Pearson's correlation between the left PF's cortical thickness and technical-reasoning performance index (TRPI) we devised ($r_{TRPI} = 0.28$, $p_{TRPI} < .01$; *Figure SR1*). The analyses of the non-normalized data confirm the same trends of the normalized data we described in the article, except for the non-normalized cortical thickness of the right area PF, which was not significant in predicting the visuospatial score ($p = 0.06$).

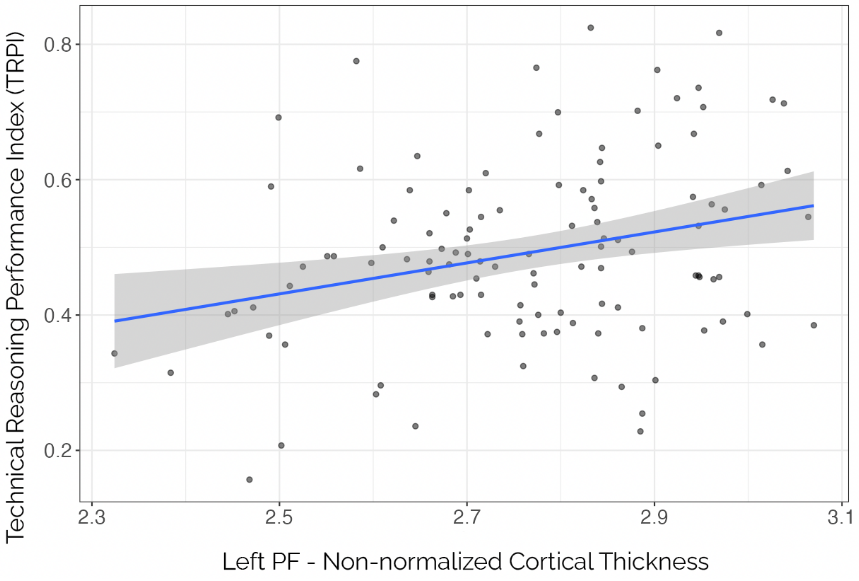

Figure SR1. The non-normalized cortical thickness of the left area PF and TRPI
Pearson's correlation between the technical reasoning performance index (TRPI) and the non-normalized cortical thickness of the area PF of the left IPC (R = .28, P < .01).

2. Cross-validation of the linear model

To further assess the weight of our main result (Left PF's Cortical Thickness → Technical Reasoning Performance), we computed k-fold cross-validation (with K = 10) of a stepwise forward model including TRPI as *dv* and all the twenty left/right IPC areas[1] as potential predictors. Therefore, with y = TRPI; $x_{(s)}$ = the cortical thicknesses of the 10 left and the 10 right IPC areas[1], the model shows that the best predictor for participants' technical-reasoning performance (TRPI) is the cortical thickness of the left area PF (R^2 = 0.24; RMSE = 0.123; *nvmax* = 1:20). To implement the analysis, we used R (v.4.1.2) and the *caret* library (https://topepo.github.io/caret).

```
nvmax  RMSE      Rsquared   MAE        RMSESD     RsquaredSD MAESD
1      0.1225591 0.23656672 0.09952959 0.02882371 0.17811576 0.02481032
2      0.1301154 0.15748942 0.10372686 0.02730268 0.13910608 0.02260033
3      0.1309477 0.14560818 0.10486131 0.02621870 0.14566573 0.02002555
[...]

Subset selection object
20 Variables (and intercept)
           Forced in Forced out
L_PF_ROI       FALSE      FALSE
L_PFt_ROI      FALSE      FALSE
L_PFop_ROI     FALSE      FALSE
[...]
1 subsets of each size up to 1
Selection Algorithm: forward
L_PF_ROI     [...]  [...]
"*"          " "    [...]
```

3. Age and gender

No effects of age and gender on technical reasoning skills are reported in the literature. However, we checked the absence of intervening factors by using an ad-hoc GLM and found no effects of gender and age in predicting participants' technical-reasoning performance.

```
Call:
lm(formula = TRPI ~ Left_PF_CT + SEX + AGE, data = raw_data, REML = TRUE)

Residuals:
     Min       1Q   Median       3Q      Max
-0.26543 -0.08595 -0.00761  0.07277  0.31769

Coefficients:
             Estimate Std. Error t value Pr(>|t|)
(Intercept) -0.506134   0.289156  -1.750 0.083001 .
Left_PF_CT   0.967129   0.263543   3.670 0.000385 ***
SEXM         0.001866   0.025314   0.074 0.941369
AGE         -0.000617   0.003048  -0.202 0.839991
---
Signif. codes:  0 '***' 0.001 '**' 0.01 '*' 0.05 '.' 0.1 ' ' 1

Residual standard error: 0.1255 on 104 degrees of freedom
Multiple R-squared:  0.1165,     Adjusted R-squared:  0.09102
F-statistic: 4.571 on 3 and 104 DF,  p-value: 0.00476

> anova (model)
Analysis of Variance Table

Response: TRPI
            Df  Sum Sq  Mean Sq F value    Pr(>F)
Left_PF_CT   1 0.21527 0.215269 13.6722 0.0003496 ***
SEX          1 0.00002 0.000019  0.0012 0.9726444
AGE          1 0.00065 0.000645  0.0410 0.8399908
Residuals  104 1.63749 0.015745
---
Signif. codes:  0 '***' 0.001 '**' 0.01 '*' 0.05 '.' 0.1 ' ' 1
```

4. Whole-brain descriptive analysis

To further verify the soundness of our findings concerning the role of the cortical thickness (CT) of the area PF in predicting technical-reasoning performance, we implemented a whole-brain descriptive analysis (*Figure SR2*). We computed a series of Pearson's correlations among the cortical thicknesses of all the brain areas (i.e., 358 regions)[1] and (i) the physical understanding score (PS); (ii) the visuospatial-imagery score (VS); (iii) the technical reasoning performance index (TRPI). We obtained R = 0.23 between PS and left PF CT, and only five higher correlations. We obtained R = 0.37 between VS and left PF CT, and no higher correlations. We obtained R = 0.21 between the VS and right PF CT, and only four higher correlations. We obtained R = 0.34 between TRPI and left PF CT, and only one higher correlation. Then, we computed the distribution of all the Pearson's correlations we obtained, which appeared normally distributed for PS, VS, and TRPI (Shapiro-Wilk W_{PS} = 0.997, Shapiro-Wilk p_{VS} = 0.793; Shapiro-Wilk W_{VS} = 0.995, Shapiro-Wilk p_{VS} = 0.231; Shapiro-Wilk W_{TRPI} = 0.995, Shapiro-Wilk p_{TRPI} = 0.286), with $R(PS)_{95th\text{-}percentile}$ = 0.157, $R(VS)_{95th\text{-}percentile}$ = 0.165, and $R(TRPI)_{95th\text{-}percentile}$ = 0.167. Therefore, given such a real data distribution, we obtained correlations concerning PF CT that are in the 95th (99th, regarding the left PF CT) percentile. The cortical thickness of the area PF seems to emerge as a potential technical-reasoning predictor not only in comparison with the other areas of the IPC but also with respect to all the brain areas[1].

	Physical Understanding	Visuospatial Imagery	TRPI
N	358	358	358
Mean	1.01e-4	8.41e-4	5.57e-4
Standard deviation	0.0971	0.0993	0.0993
Minimum	-0.271	-0.295	-0.255
Maximum	0.281	0.370	0.362
Shapiro-Wilk W	0.997	0.995	0.995
Shapiro-Wilk p	0.793	0.231	0.286
95th percentile	0.157	0.165	0.167

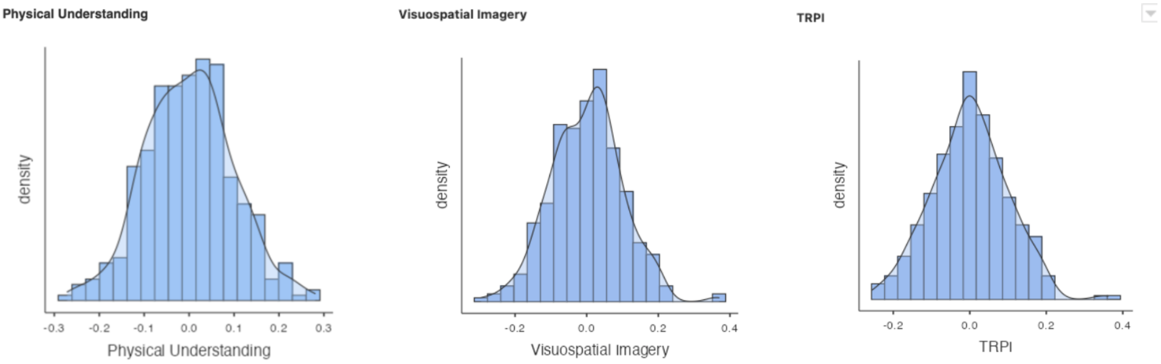

Figure SR2. Whole-brain descriptive analysis

References

1. Glasser, M. F. *et al.* A multi-modal parcellation of human cerebral cortex. *Nature* **536**, (2016).
2. Bernaud, J.-L., Priou, P. & Simonnet, R. *Batterie multifactorielle d'aptitudes [Multifactor Aptitude Battery]*. (Editions du Centre de Psychologie Appliquée, 1994).
